# Quantitative analysis of receptor-mediated uptake and pro-apoptotic activity of mistletoe lectin-1 by high content imaging

**DOI:** 10.1038/s41598-018-20915-y

**Published:** 2018-02-09

**Authors:** N. Beztsinna, M. B. C. de Matos, J. Walther, C. Heyder, E. Hildebrandt, G. Leneweit, E. Mastrobattista, R. J. Kok

**Affiliations:** 1 Department of Pharmaceutics, Utrecht Institute for Pharmaceutical Sciences, Utrecht University, Utrecht, The Netherlands; 2 ABNOBA GmbH, Pforzheim, Germany; 3 Institute for Mechanical Engineering and Mechanics, Karlsruhe Institute of Technology, Karlsruhe, Germany

## Abstract

Ribosome inactivating proteins (RIPs) are highly potent cytotoxins that have potential as anticancer therapeutics. Mistletoe lectin 1 (ML1) is a heterodimeric cytotoxic protein isolated from European Mistletoe and belongs to RIP class II. The aim of this project was to systematically study ML1 cell binding, endocytosis pathway(s), subcellular processing and apoptosis activation. For this purpose, state of the art cell imaging equipment and automated image analysis algorithms were used. ML1 displayed very fast binding to sugar residues on the membrane and energy-dependent uptake in CT26 cells. The co-staining with specific antibodies and uptake blocking experiments revealed involvement of both clathrin-dependent and -independent pathways in ML1 endocytosis. Co-localization studies demonstrated the toxin transport from early endocytic vesicles to Golgi network; a retrograde road to the endoplasmic reticulum. The pro-apoptotic and antiproliferative activity of ML1 were shown in time lapse movies and subsequently quantified. ML1 cytotoxicity was less affected in multidrug resistant tumor cell line 4T1 in contrast to commonly used chemotherapeutic drug (ML1 resistance index 6.9 vs 13.4 for doxorubicin; IC_50_: ML1 1.4 ng/ml vs doxorubicin 24000 ng/ml). This opens new opportunities for the use of ML1 as an alternative treatment in multidrug resistant cancers.

## Introduction

Ribosome inactivating proteins (RIPs) are highly potent cytotoxins that interfere in protein biosynthesis. RIPs have been found and isolated from various natural resources such as plants, bacteria, fungi and algae. Plant derived RIPs play an important role as defense against herbivores^1,2^. From clinical point-of-view, RIPs are considered as anticancer therapeutics^3,4^. The large RIP family comprises all enzymes EC 3.2.2.22 that catalytically inactivate eukaryotic protein synthesis by hydrolyzation of the N-glycosidic bond between adenine-4324 and the nucleotide in the 28 S rRNA of the 60 S subunit of ribosomes. The rRNA is fragmented and it ultimately results in protein synthesis inhibition^1,2,5–10^ and caspase-mediated apoptosis and necrosis^8^. Toxic RIPs act at very low doses (less than equimolar ratio to the substrate) since the inactivation of ribosome protein production is irreversible^1,2,5–10^.

RIPs can be generally classified in three groups. The class of monomeric ~30 kDa RIP-I contains an enzymatic chain only. The class of heterodimeric ~60 kDa RIP-II cytotoxins has the enzymatic chain linked to a lectin chain, often referred as B-chain. The B-chain has high affinity for sugar moieties on the cells surface which promotes protein binding and mediates the protein uptake^1,2,5–9,11^. Due to the absence of a lectin chain, RIP-I do not internalize as efficiently as RIP-II and some of them are considered relatively safe^9^. Specific cell binding ligands could be conjugated to RIP-I to increase the therapeutic value. In addition to these two classes, RIP type III cytotoxins consist of a toxic unit linked to a peptide with unknown function^10^.

Ricin and Abrin are among the best well studied plant derived RIP-II cytotoxins. Mistletoe lectin 1 (ML1) is also categorized as RIP-II and it is one of the main active components of *Viscum album* extracts. Although *Viscum album* extracts are used as an adjuvant in alternative medicine practices^12,13^, there is a lack of scientific understanding of how ML1 as a major extract component contributes to the perceived gain in quality of life, and thus the human-beneficial potentialities might be underestimated or misunderstood.

Scientific reports on ML1 are mostly referring to Korean ML or recombinant variations of ML1. Reports on European ML1 are however scarce. Although European ML1 shares 84% homology with Korean ML^14–19^, it cannot be assumed they have the same subcellular modes of action and pathways; the same is true for recombinant variations of mistletoe lectin or plant extracts containing ML1^20–23^.

ML extracts as well as isolated and recombinant versions of the protein have shown potent cytotoxic activity against tumor cells *in vitro*^21,24–27^ as well as has contributed to prolonged cancer-free survival in some clinical studies^12,28–30^. Interestingly, ML1 was pointed out as a suitable candidate for treatment of breast cancer in clinical and preclinical trials but despite the promising initial results, no follow up research was done^31,32^. Other studies have suggested that ML1, especially the B-chain, has an immunomodulatory activity^21,33,34^. However, this information is sometimes contradictory and/or lacking proper controls. The mechanisms of ML1 uptake and subsequent cell processing are often equated in the literature to other similar toxins such as ricin or Shiga toxin^35–38^; but direct data on the cellular fate of ML1 is limited and most of the studies were performed on paraformaldehyde pre-fixed cells^36,37,39^.

Our aim was to take the first steps towards a better understanding of the uptake mechanism and cytotoxicity of European ML1. We investigated in detail ML1 binding, uptake pathways and endosomal escape mechanisms in correlation with its cytotoxic activity in living cells. For this purpose, state-of-the-art high content imaging was used in combination with advanced image analysis algorithms. The present study sheds light on the ML1 mechanism of action that can be helpful for cancer treatment, but also provides advanced methodological tools for the investigation of the interaction of proteins, macromolecules or nanoparticles with cells in real time with high speed and accuracy.

## Results and Discussion

### ML1 isolation and labeling

**Figure 1:**
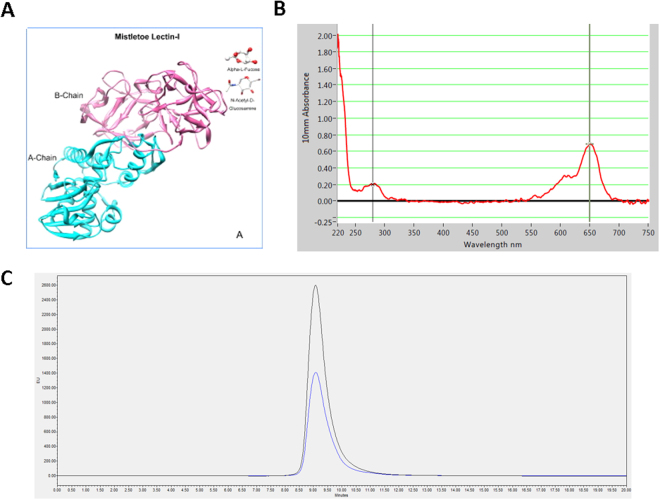
(**A**) Mistletoe lectin 1 (ML1) structure^53^; (**B**) Absorbance spectrum of AF647 labeled ML 1; (**C**) GPC chromatogram of AF647 labeled ML1 (absorbance at 280 nm –blue, at 650 nm – black).

Mistletoe lectin 1 is composed of a cytotoxic A chain and a galactose binding B chain (Fig. 1A). It shares 52% sequence homology with ricin, one of the most common RIP-II^40^. ML1 was extracted and isolated from plant material as described previously^41^. characterized with FPLC and PAGE (Supplementary information, Figure S1) and subsequently labeled with Alexa Fluor 647 NHS (AF647) via covalent conjugation. ML1 – AF647 conjugates (further abbreviated as ML1, Fig. 1B) were purified and analyzed by size exclusion chromatography (Fig. 1C). The fluorophore to protein molar ratios after conjugation were around 1:1. The activity of fluorophore conjugated ML1 was compared to the native protein and no significant differences were found (Supplementary information, Figure S2). To exclude non-specific uptake of labeled proteins, AF647 was conjugated to bovine serum albumin (BSA) as a negative control. As expected, there was no uptake of this conjugate in CT26 cells after 1 h incubation (data not shown).

### ML1 uptake in CT26 cells

**Figure 2:**
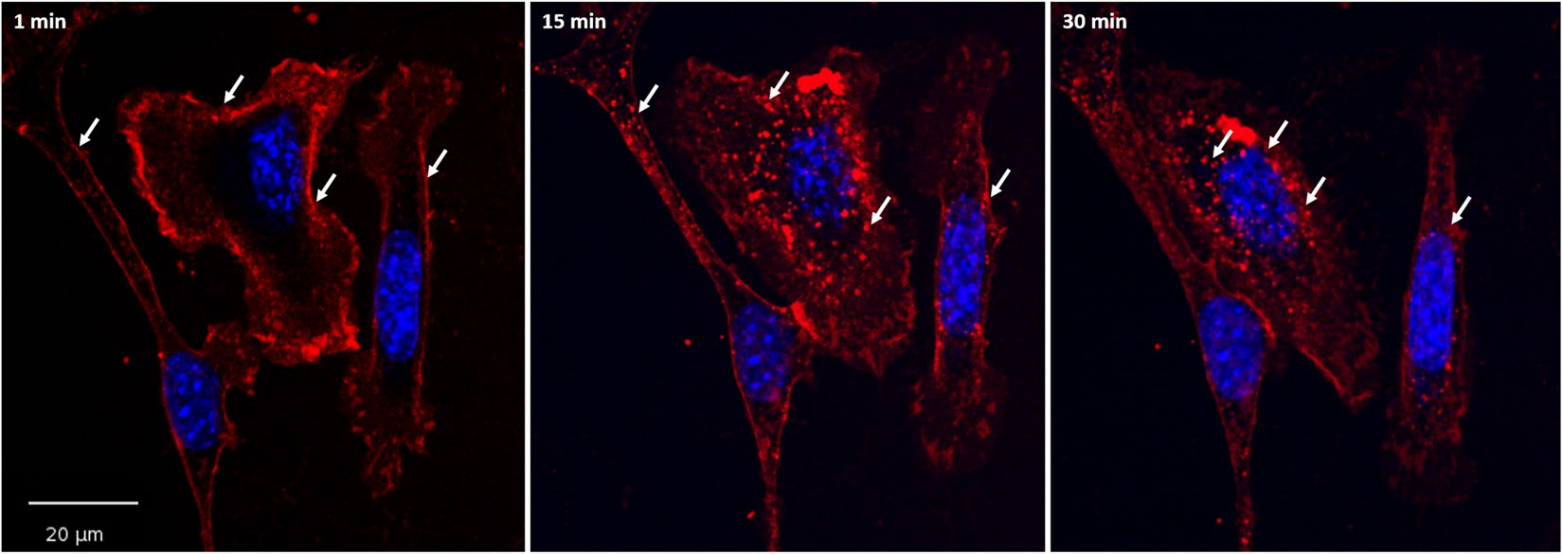
Confocal images of ML1 uptake in CT 26 cells at early time points; nuclei are stained in blue, ML1 – red; arrows indicate changes in the uptake patterns from membrane binding to vesicles that move closer to the nuclei in time; size bar 20 µm.

The uptake of ML1 conjugates was examined in living murine colon carcinoma cells (CT26) by fluorescence confocal microscopy using the Yokogawa Cell Voyager CV7000s (Fig. 2). Their murine origin makes CT26 tumor cells especially suitable for use in cancer models in immunocompetent mice, which are more representative of clinical cancer than xenograft models in nude athymic mice. The CV7000s imaging platform consists of an automated confocal laser-scanning microscope placed in the environmental chamber therefore allowing the acquisition of live cell images in real time. The high speed of auto-focusing and image acquisition allowed capturing the earliest steps of ML1 binding. As seen on the Fig. 2, the protein immediately binds to cells outlining their membrane on the microscopy image. The same binding pattern is observed when cells are incubated with ML1 at 4 °C and fixed (Supplementary Information, Figure S3). As early as 15 minutes after addition of the protein to cells, ML1 appears in cytoplasmic vesicular structures and their number and fluorescence intensity increases over time. Moreover, at later time points these vesicles seem to get closer to the perinuclear region.

### Comparison of ML1 uptake with other toxins

**Figure 3:**
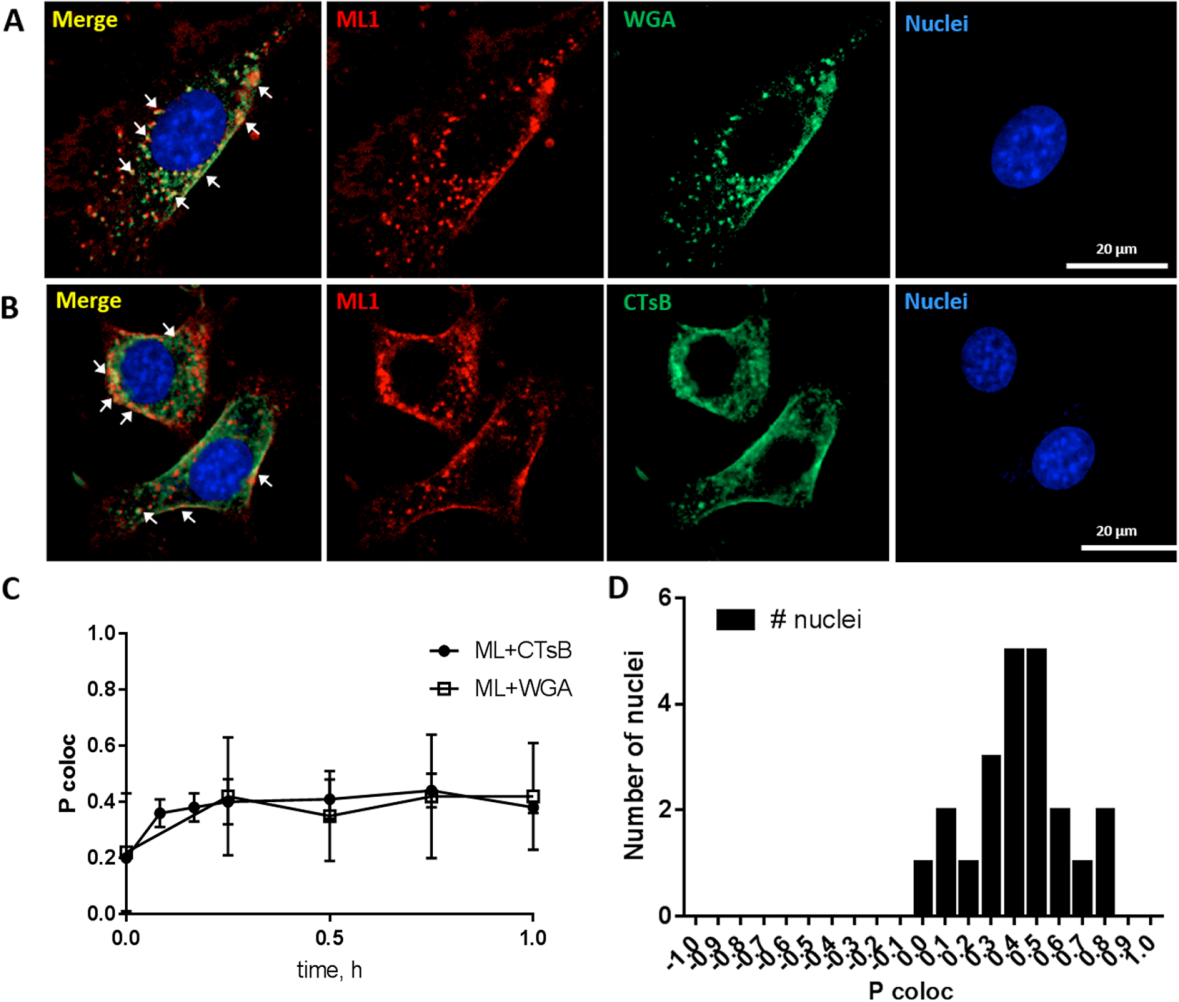
Confocal images of ML1 (in red) co-localization with: (**A**) WGA (in green); (**B**) CTsB (in green). (**C**) Pearson correlation coefficient calculated with Columbus Suit software (P coloc) of ML1 co-localization with WGA or CTsB at various time points. (**D**) Frequency distribution of P coloc between ML1 and CTsB after 1 h incubation; CT 26 cells, nuclei are stained in blue; size bar 20 µm.

The binding and uptake of ML1 in CT26 cells was simultaneously compared to Oregon Green labeled Wheat Germ Agglutinin (WGA) and Alexa Fluor 488 labeled Cholera Toxin subunit B conjugate (CTsB). All three proteins displayed very similar uptake pattern with membrane binding in first minutes followed by location in vesicles in the cytoplasm (Fig. 3A,B).

The co-localization of ML1 and either of the toxins in intracellular vesicles was quantified with the help of Columbus Suit software calculating Pearson correlation coefficient (P coloc). P coloc is a well-established measure of correlation and ranges from +1 (perfect correlation) to −1 (perfect negative correlation) with values close to 0 meaning an absence of correlation. For the two fluorophores P coloc measures the correlation between their intensities in a given picture and is meant to reflect on a molecular interaction between molecules or with subdomains of a cellular compartment^42^. Columbus co-localization algorithm P coloc calculation provided correlation plots in a faster and more automated manner than classical image analysis tools such as Image J Software (Coloc 2 tool). Both software tools were applied on the same dataset (ML1 and CTsB after 1 h of co-incubation) and yielded almost identical P coloc values 0.44 ± 0.13 and 0.45 ± 0.15 for Image J and Columbus, respectively.

Co-localization analysis in time showed similar degree of association between ML1 and WGA or ML1 and CTsB (Fig. 3C,D). Figure 3D shows an example of P coloc distribution analysis in one of the images of ML1 and CTsB after 1 h of co-incubation. The Pearson’s correlation coefficient varied from 0.2–0.3 at the first minutes of experiment (membrane binding step) to 0.4–0.6 after 15–60 minutes of continuous incubation with both protein pairs. Although theoretical P coloc maximum is 1, the correlation of 0.5 and higher is considered high enough to show the co-localization in biological systems^43,44^. The observed P coloc values indicate high degree of similarity between ML1, WGA and CTsB in terms of cellular uptake, although their targeted receptors on the cell surface are different^45^. CTsB is frequently used in the literature as a marker of lipid-raft associated caveolin-mediated uptake while it was also shown to enter cells through clathrin-dependent pathway and clathrin- and caveolin- independent pathways^46,47^.

### Inhibition of ML1 uptake

Various uptake inhibitors were applied to elucidate the key mechanisms involved in ML1 uptake by the cells. The results are based on microscopic observations and subsequent quantitative image analysis (Supplementary Figures S4 and S5). Incubation of the cells with ML1 at 4 °C resulted in binding to the outer membrane surface without any observable intracellular signal (Supplementary information, Figure S2). This result suggests that ML1 uptake is energy-dependent. The molecular target for ML1 on the cell surface is a specific polysaccharide ligand containing a terminal sialic acid residue and D-galactose. Non-surprisingly, the pre-incubation of cells with excessive amounts of galactose (10–100 mM) inhibited both uptake and membrane binding of ML1. It was previously reported that high amounts of galactose in the cell medium could protect cells from ML1 associated toxicity^48^. Inhibitors of clathrin-dependent uptake, chlorpromazine and high concentration of sucrose, noticeably decreased ML1 uptake and binding but did not inhibit it completely suggesting the involvement of clathrin-independent mechanism in ML1 uptake. Finally, methyl-β-cyclodextrin, a cholesterol-depleting agent that abolishes caveolae mediated endocytosis^49^, did not show any influence on ML1 membrane binding and uptake.

### ML1 co-localization with subcellular compartments

**Figure 4:**
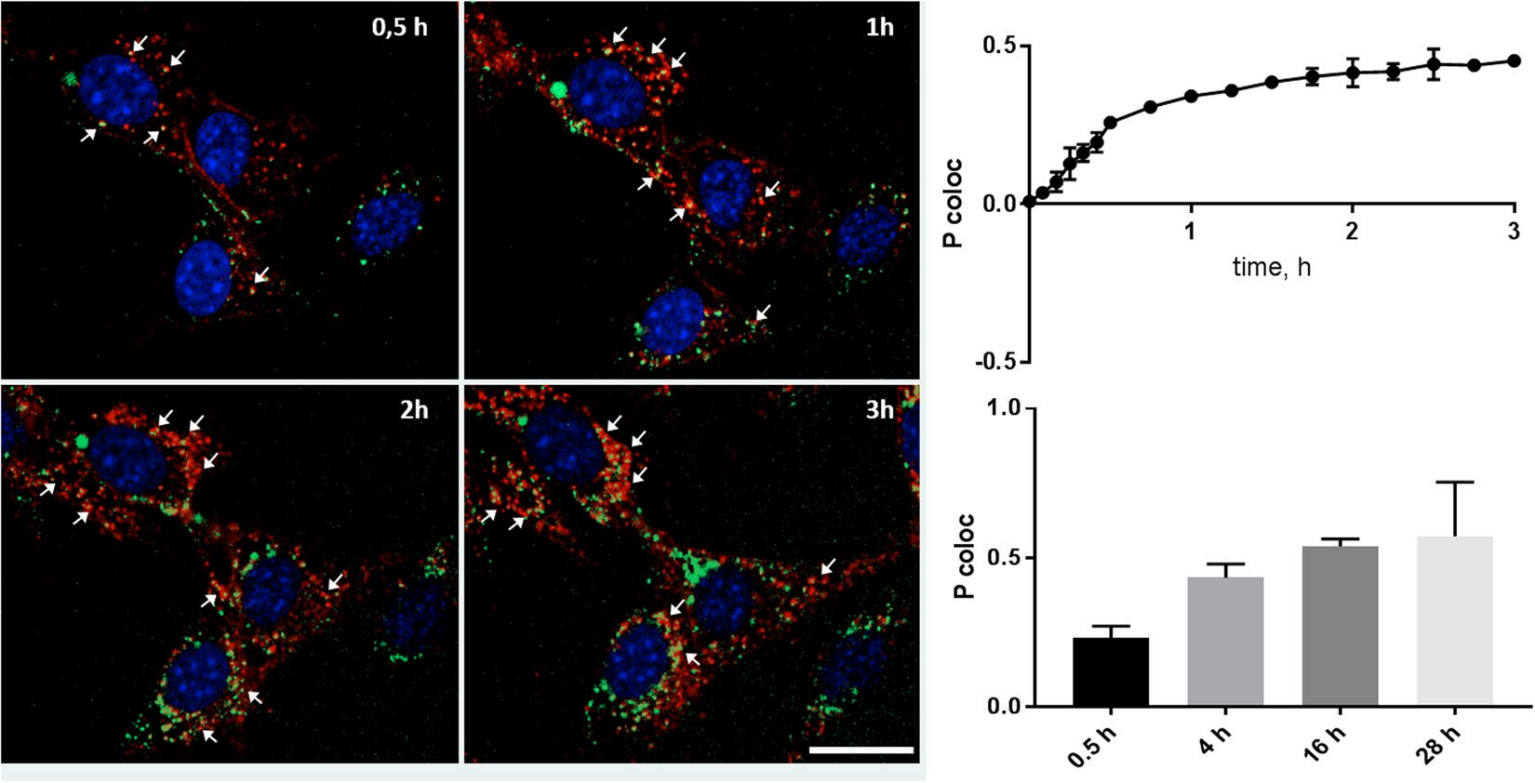
Confocal images of ML1 (in red) co-localization with endosomal marker (pHrodo™ Green Dextran, in green) at different time points (left) and Pearson correlation coefficient dynamics of co-localization ML1 with endosomes at early (up, right) and late time points (lower, right). CT 26 cells, nuclei are stained in blue; arrows indicate co-localization regions; size bar – 20 µm.

The dynamic accumulation of ML1 in various subcellular compartments was studied by coincubation with various markers that are compatible with microscopy of living cells (summarized table of all used cell markers is presented in Supplementary Information, Table S1). CT26 cells were first pre-incubated with endosomal, lysosomal and Golgi vesicular markers in separate experimental setups (pHrodo™ Green Dextran was used for endosomes, LysoTracker® Green for lysosomes and BODIPY® TR C5-ceramide complexed to BSA for Golgi). Then the cells were incubated with 10 µg/mL of ML1 and imaged. Figure 4 shows gradual increase in time in the overlapping between the green signal from endosomes (pHrodo™) and red signal from ML1. Co-localization analysis at short (up to 3 h, and long (up to 28 h) kinetics revealed time-dependent increase in correlation of intracellular ML1 signal with endosomal labelling (Fig. 4).

**Figure 5:**
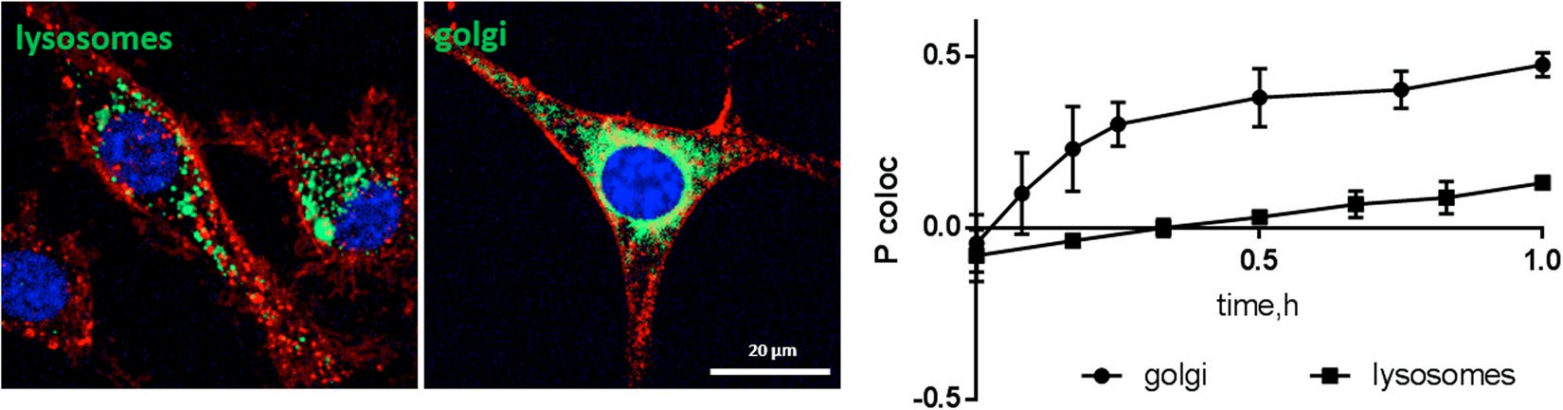
Confocal images of ML1 (in red) co-localization with lysosomal or Golgi markers (in green) after 1 h incubation and Pearson correlation coefficient dynamics of co-localization ML1 with lysosomes and Golgi. CT 26 cells, nuclei are stained in blue; arrows indicate co-localization regions; size bar – 20 µm.

Co-localization analysis of ML1 with lysosomal marker (LysoTracker® Green) revealed little to no correlation (P coloc around 0, Fig. 5). In contrast, Golgi apparatus labeling with BODIPY® TR C5-ceramide complexed with BSA showed substantially higher co-localization with intracellular ML1 already at 30 min incubation (P coloc 0.38 for Golgi versus 0.03 for lysosomes). This indicates that ML1 does not go through the endolysosomal pathway leading to protein degradation, but instead follows a retrograde pathway from endosomes to Golgi and endoplasmic reticulum as previously shown for ricin, Shiga toxin and Cholera toxin^50^.

**Figure 6:**
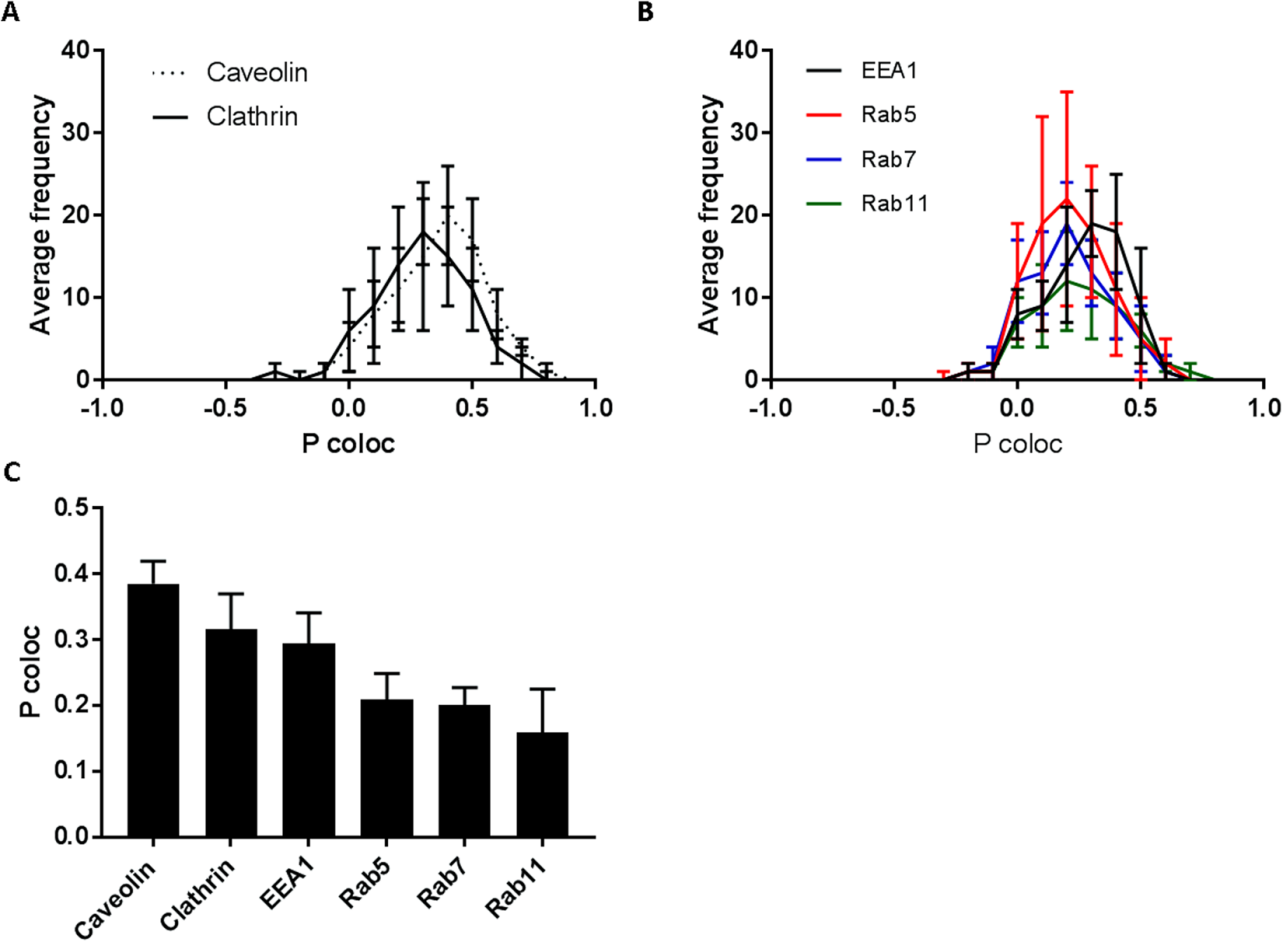
Co-localization analysis of ML1 and endosomal pathway specific antibodies: (**A**) Pearson’s coefficient frequency distribution of ML1 co-localization with caveolin or clathrin specific antibodies; (**B**) Pearson’s coefficient frequency distribution of ML1 co-localization with early, late endosome or lysosome specific antibodies; (**C**) Median Pearson’s coefficient of ML1 and endosomal pathway specific antibodies co-localization. CT26 cells were incubated with 10 µg/mL of ML1 for 2 h, then fixed, permeabilized and stained with specific antibodies.

To confirm live imaging data, ML1 uptake was analyzed with a set of endolysosomal pathway specific antibodies (Fig. 6, Supplementary information, Figure S6). CT26 cells were incubated with ML1 for 2 h, fixed and then stained with anti EE1 and anti Rab5 for early endosomes, anti Rab7 for late endosomes, anti Rab11 for lysosomes, anti-clathrin and anti-caveolin. Subsequent co-localization and statistical analysis revealed the ML1 association with both clathrin and caveolin staining with slight preference towards the former. This is in accordance with the uptake inhibition experiments and the co-localization data described above, indicating a progressively decreasing amount of ML1 associated with later stages of endolysosomal pathway (Rab5, Rab7 and Rab11) and thus confirming its redirection towards Golgi and retrograde transport to the ER. The exact “endosomal escape” mechanism remains unclear for plant derived toxins in general and for ML1. Ricin toxin, shares a lot of similarities with ML1, induces membrane destabilization and structural changes at low pH (fusion and lysis), which allows it to escape endocytic vesicles and undergo retrograde transport to the ER with subsequent translocation to the cytoplasm^51,52^.

### ML1 cytotoxicity

**Figure 7:**
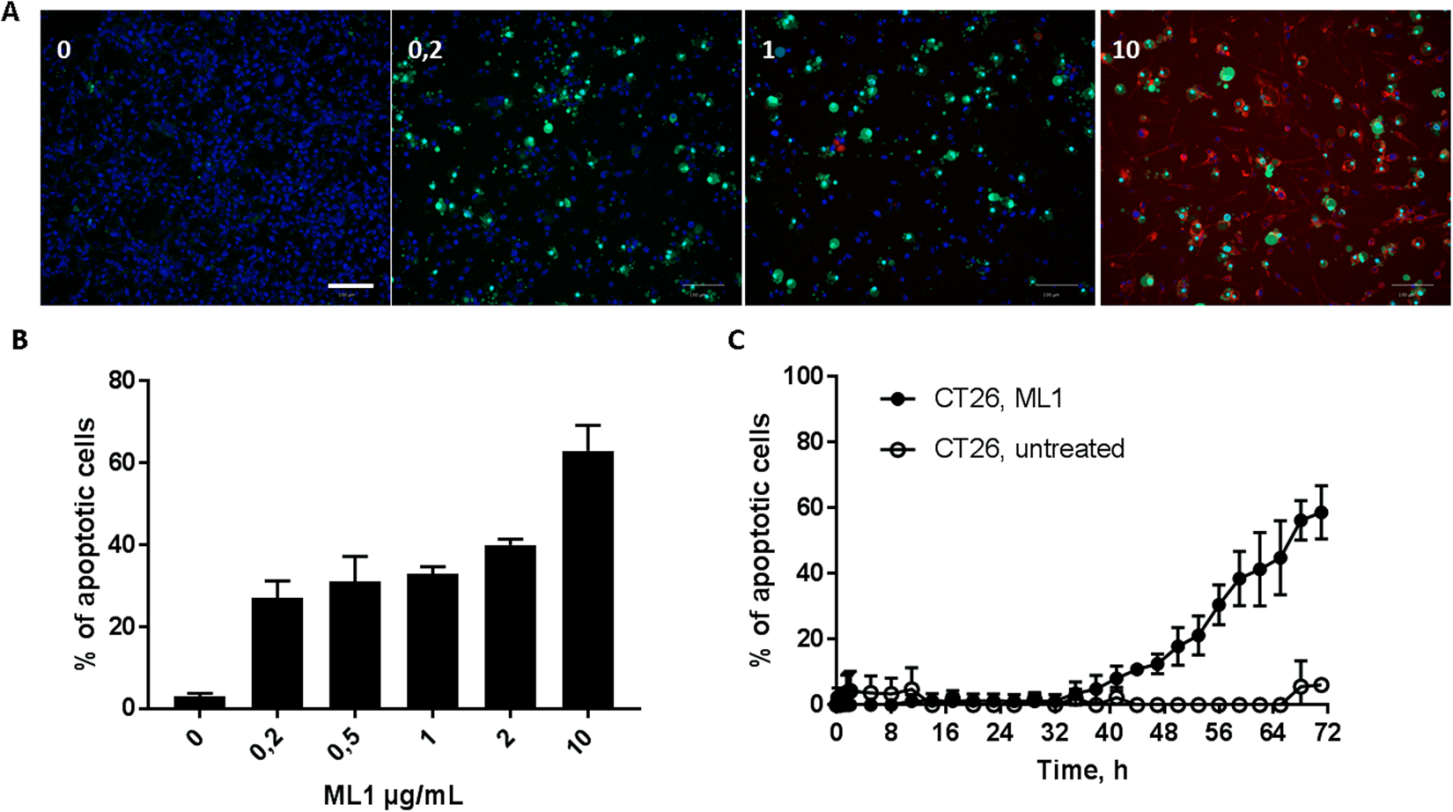
(**A**) Confocal images of apoptosis in CT 26 cells after 71 h incubation with ML1 at different concentrations; nuclei are stained in blue, apoptotic cells in green (CellEvent® live staining), ML1 in red; size bar 100 µm. (**B**) CT 26 cell viability after 71 h incubation with different concentrations of ML1; (**C**) Percentage of apoptotic cells at different time points during incubation with 10 µg/mL of ML1.

**Figure 8:**
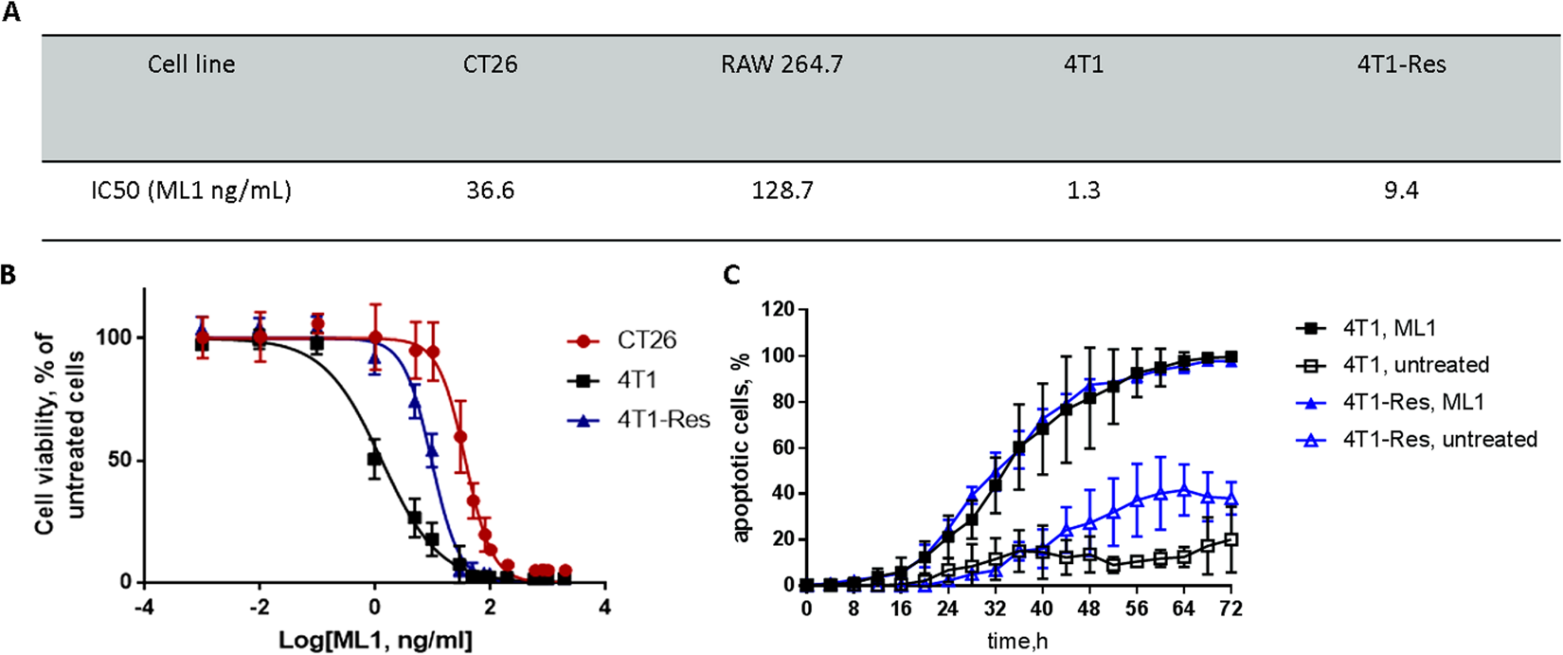
(**A**) Comparative cytotoxicity of ML1 in different cell lines. (**B**) IC_50_ curves for CT26, 4T1 parental and 4T1 Dox Resistant cell lines; (**C**) Percentage of apoptotic cells at different time points during incubation of 4T1 parental and 4T1 Dox Resistant with 200 µg/mL of ML1. Non-treated cells were used as negative control.

Pro-apoptotic and anti-proliferative activity of ML1 were demonstrated in viability and caspase activation assays in live-cell imaging (Figs 7 and 8). ML1 demonstrated a dose-dependent induction of apoptosis in CT26 cells through caspase activation mechanism. The latter was determined by CellEvent™ green pan caspase activation marker. It consists of nucleic acid binding dye coupled to a fluorescence quenching peptide. In healthy cells CellEvent™ can be freely taken up but is not fluorescent. But if caspases 3/7 or 8 are present in the cell, quenching peptide is cleaved, the dye binds to DNA in the nuclei and displays bright green fluorescence. In this way, early apoptosis could be detected and quantified by image analysis tools. Moreover, the assay itself is not toxic to the cells and could be monitored continuously over time with live imaging (Fig. 7, Supplementary information, movies 1 and 2).

Even though the ML1 uptake and translocation towards retrograde Golgi transport were very fast (minutes to hours), the caspase mediated apoptosis activation was detectable much later, after at least one day (Fig. 7, Supplementary movies 1 and 2). The total cell number was visibly lower in ML1 treated cells indicating not only pro-apoptotic but also anti-proliferative activity of ML1 (Fig. 7A). In the literature ML1 was shown to activate apoptosis though various pathways such as TNF-alpha induction, external caspase 8 activation as well as Bax and Bad proteins translocation to the mitochondria^53^.

The anti-proliferative activity of ML1 was investigated in additional cell lines (Fig. 8A). Interestingly, the IC_50_ of studied murine cancerous cell lines was 3.5 to 100-fold lower compared to murine inflammatory cell line RAW264.7. These differences could be potentially attributed to variations in cell glycosylation patterns, which results in lower or higher sensitivity to ML1^26^. The altered glycosylation pattern was also shown to influence multidrug resistant tumor cell’s sensitivity to ML1^26^. Experiments performed using a chemoresistant variant of the murine triple negative (TNBC) breast cancer cell line 4T1 are represented on Fig. 8A,B. When compared to the reported values of doxorubicin^43^, a standard chemotherapeutic for treatment of breast cancer patients, ML1 was 2 × less affected by antineoplastic resistance (ML1 resistance index 6.9 vs 13.4 for doxorubicin). Furthermore, ML1 is almost 4 orders of magnitude more cytotoxic than doxorubicin in the chemo-sensitive cell line (IC_50_: ML1 1.4 ng/ml in Fig. 8A, as compared to doxorubicin 24000 ng/ml reported by Chen *et al*.^43^). Real-time apoptosis imaging revealed a very similar pattern to the one observed for CT26 cells (Fig. 8C). The slightly higher spontaneous apoptosis rate in non-treated chemoresistant 4T1 cells may be due to increased expression levels of caspases or nonspecific proteases that will generate false positive signals in the CellEvent™ assay (Supplementary information, movies 3 and 4). Nevertheless, the subtraction of spontaneous caspase activation in non-treated 4T1 Res cells from ML1 incubated still gives the net increase in apoptosis rate of around 60%, which is comparable to 53% for CT26 cells reported Fig. 7C. Even though the IC_50_ of sensitive and drug resistant cells was different, the ML1 uptake profile and its co-localization with cell organelles were very similar for both cell lines (Supplementary information, Figure S7).

## Conclusions

The present study clearly demonstrated crucial steps for the uptake of isolated ML1: i. glycan binding on the cell surface; ii. clathrin-dependent and -independent endocytosis; iii. redirection of the protein from endocytic vesicles to Golgi network and presumably its subsequent retrograde transport to the ER. These steps are crucial for the protein to reach its molecular target in the cytosol and ensure anti-proliferative and pro-apoptotic activity. In this context, ML1 is a potential cytotoxin which can be applied for eradication of multidrug resistant tumor cells which respond poorly to anticancer drugs that are transported via ABC transporters (including P-glycoprotein [P-gp] and multi-drug resistance proteins). While those mechanisms alter the performance of conventional chemotherapeutic drugs (taxanes, platinum drugs etc.), ML1 might overcome the resistance due to a specific uptake pathway, subcellular processing and sorting.

## Materials and Methods

### Materials

All cell labeling reagents and fluorescent dyes were purchased from Thermo Fisher Scientific, Naarden, The Netherlands unless stated otherwise. Cell medium and supplements were from GibcoBRL, Thermo Fisher Scientific, Naarden, The Netherlands.

### Methods

#### Purification and characterization of Mistletoe lectin I

For ML1 isolation, mistletoe plant material was harvested in June from ash tree (Fraxinus excelsior L.) and extraction was performed by affinity chromatography as described previously^41^, using the affinity of ML1 for D-galactose. After purification, ML-1 was characterized by FPLC using a Mono S cation exchange column (Pharmacia/GE Healthcare, Uppsala, Sweden) and a 0.6 M NaCl salt gradient in 0.015 M citrate buffer (pH 4.0) at a detection wavelength of 280 nm (Supplementary information, Figure S1A). Further characterization was performed by non-denaturing and denaturing SDS-PAGE to confirm the size of native ML-1, and presence of the A and B chains (12% PAA, 1 h at 160 V, LMW SDS Marker; GE Healthcare, Munich, Germany). For non-denaturing SDS-PAGE, ML was pretreated with saturated iodoacetamide solution (1:2) for 30 min at RT to prevent autolytic cleavage of the single disulfide bond which covalently links A and B chains. Gels were stained using Coomassie blue (Supplementary information, Figure S1B). ML-1 was quantified at 280 nm using an extinction coefficient of 1.41 and by ELISA using monoclonal (5F5) and POD-labelled polyclonal (5H8) anti-ML A chain antibodies (Sifin Diagnostics GmbH, Berlin, Germany).

#### ML1 fluorescent tagging

ML1 conjugation to Alexa Fluor 647 (AF647) succinimidyl ester was performed in accordance to manufacturer protocol. In brief, the protein was dissolved in PBS buffer at 5.6 mg/mL and then diluted with 0.02 M bicarbonate buffer (2000 + 250 µL) to ensure basic pH. The 2250 µL of diluted protein were added to aliquoted dye (100 µg) diluted in 20 µL of anhydrous DMSO. The mixture (protein to dye molar ratio – 1:5) was stirred at room temperature for 1 h. The protein-dye conjugate was purified with a PD10 size exclusion column and then analyzed with GPC (Bio Sep 3000 column, 20 min, PBS 1 mL/min). The labeled protein stock solution was filtered through 0.45 µm syringe filter and protein concentration was quantified by absorbance at 280 nm with NanoDrop® spectrophotometer (Isogen Life Sciences B.V., Utrecht, The Netherlands).

### Cell culture

The murine colon carcinoma cells (CT26 WT) were obtained from American Type Culture Collection (ATCC) and cultured in RPMI-1640 medium (Sigma Aldrich) supplemented with 10% FBS (Sigma Aldrich). The murine breast cancer cells (4T1) – mother and doxorubicin resistant cell lines were kindly provided by Prof. Twan Lammers from Uniklinik RWTH Aachen, Germany and cultured in RPMI-1640 medium (Sigma Aldrich) supplemented with 10% FBS (Sigma Aldrich). The murine macrophages (RAW 264.7) were also obtained from ATCC and cultured in DMEM (Sigma Aldrich) supplemented with 10% FBS (Sigma Aldrich). All cells were maintained at 37 °C in a 5% CO_2_ and humidified atmosphere.

#### Organelle labeling

All subcellular structures labeling was performed according to the manufacturer instructions. For the nuclei staining, the cells were incubated with 10 nM Hoechst 33342 in PBS for 10 min at 37 °C. Lysosomes were stained with 75 nM LysoTracker Green solution in PBS for 30 min at 37 °C. Endosomes were labeled with pHrodo Green Dextran conjugate diluted in complete growth medium to a final concentration of 0.05 mg/mL for 20 min at 37 °C. For Golgi staining cells were first incubated on ice with cold solution of BODIPY® TR C5 -ceramide complexed to BSA at final concentration of 5 µM, then washed with cold PBS and incubated with fresh medium for further 30 min at 37 °C. Summarized table of all used cell markers is presented in Supplementary Information, Table S1.

#### ML1 uptake

Cells were seeded into 96-well µClear^®^ black plates (Greiner, 10000 cells/ well) and incubated overnight. Then, medium was replaced with fresh complete medium and appropriate amount ML1-AF647 conjugate (ML1, from 0.0002 to 20 µg/mL) was added. For live imaging experiments the cells were placed in the microscope and imaged immediately with CV7000s and then as often and as long as it was required for a particular experiment. For endpoint imaging, the cells were incubated with ML1 for desired time, then washed with PBS and fixed with 10% formaldehyde solution (30 min at room temperature). The uptake at low temperatures was performed with pre-cooled cells (15 min at 4 °C), cold medium and PBS. The cells were incubated with cold solution of ML1 (10 µg/mL) for 1 hour, then washed and fixed with 10% formaldehyde solution as described above.

#### WGA and cholera toxin subunit b uptake

Cells were seeded into 96-well µClear^®^ black plates (Greiner, 10000 cells/well) and incubated overnight. The medium was replaced with fresh complete medium and Wheat Germ Agglutinin Oregon green or Cholera toxin subunit b. Alexa Fluor 488 conjugates were added to the cells to a final concentration of 5 and 10 µg/mL, respectively. In co-localization experiments 10 µg/mL of ML1-AF647 conjugates were added to the cells as well. The cells were imaged immediately with CV7000s.

#### Inhibition of ML1 endocytosis

For the uptake inhibition experiments cells were seeded into 96-well µClear^®^ black plates (Greiner, 10000 cells/ well) and incubated overnight. Next day the medium was replaced with fresh full growth medium containing appropriate concentration of the inhibitor and cells were pre-incubated for 15–30 min (galactose 10–100 mM, sucrose 0,4 M, chlorpromazine 12.5–100 µM, methyl-β-cyclodextrin 10–250 mM). Then 10 µg/mL of ML1-AF647 conjugates were added to the cells and after 1 h incubation cells were imaged with CV7000s and ML1 uptake was quantified. Non-treated cells were used as positive uptake control. For the low temperature experiments, cells were first washed with ice-cold medium, pre-incubated 15 min at 4 °C and then incubated with 10 µg/mL of ML1-AF647 conjugates at 4 °C for 1 h. After incubation, cells were washed twice with ice-cold PBS and fixed with 4% PFA solution (10 min at room temperature). After fixation, cells were washed twice with PBS and imaged with CV7000s. The same procedure was performed for the control plate that was incubated at 37 °C. To facilitate subsequent image analysis, prior imaging cell nuclei were labeled with Hoechst 33342 as described above.

#### Endosomal pathways labeling with antibodies

Endosomal Marker Antibody Sampler Kit #12666 was purchased from Cell Signaling Technology Europe, B.V. (Leiden, The Netherlands) and used according to manufacturer instructions. Briefly, cells were seeded into 96-well µClear^®^ black plates (Greiner, 10000 cells/well) and incubated overnight. Then the medium was refreshed with full complete growth medium and 10 µg/mL of ML1-AF647 conjugates were added to the cells. After 2 h incubation cells were washed twice with PBS and fixed with 4% PFA solution (10 min at room temperature). Cell nuclei were labeled with Hoechst 33342 as described above. Then cells were pre-incubated with blocking buffer (5% FBS, 0,3% Triton in PBS) for 1 h. Antibodies were diluted in antibody dilution buffer (1% BSA, 0,3% Triton in PBS) as following: anti-caveolin 1:400, anti-clathrin 1:50, anti-EEA1 1:100, anti-Rab5 1:200, anti-Rab7 1:100 and anti-Rab11 1:100. Cells were incubated with diluted antibodies overnight at 4 °C, then washed 3 times with PBS and incubated with secondary antibody (Anti-Goat conjugated to Alexa Fluor 488, dilution 1:100) for 2 h at room temperature. Finally, cells were washed twice with PBS and imaged with CV7000s.

#### Cytotoxicity assays

Cells were seeded in 96 well plates (CT26 and 4T1 10000 cells/well; RAW264.7 5000 cells/well) and left to adhere for 24 h. Various concentrations of ML1 (2 µg/ml–0.001 ng/ml) were incubated with cells for 48 h, after which cell survival was determined by MTS conversion (CellTiter 96® AQueous One Solution Cell Proliferation Assay) at 490 nm in a well plate reader. IC_50_ (the concentration of drug causing 50% reduction of the survival of the control) was calculated from the survival growth curves by fitting using GraphPad Prism software.

#### Apoptosis induction detection

To investigate the caspase-mediated pro-apoptotic activity of ML1 commercially available CellEvent™ caspase-3/7 Green Detection Reagent was used. Cells were seeded into 96-well µClear® black plates (Greiner, 10000 cells/ well) and incubated overnight. First, cell nuclei were labeled with Hoechst 33342 as described above. 30 min before the experiment, cell medium was refreshed with full complete growth medium containing 10 µM of CellEvent™ Caspase-3/7 Green Detection Reagent. Then various concentrations of ML1-AF647 conjugates (200–0.0002 µg/mL) were added. The cells were imaged immediately with CV7000s with images taken every 4 h for up to 72 h in total.

### Image analysis

Customized image analysis protocols were developed with Columbus Software (U.S. National Institutes of Health, Bethesda, Maryland, USA).

## Electronic supplementary material

Supplementary information

Movie 1 CT26 Apoptosis assay 20 ug mL ML1 0–72h 60x

Movie 2 CT26 Apoptosis assay control ML1 0–72h 60x

Movie 3 4T1 Res Apoptosis assay 2 ug mL ML1 0–72h 40x

Movie 4 4T1 Res Apoptosis assay control 0–72h 40x
