# Supplementary material for: Quantitative analysis of receptor-mediated uptake and pro-apoptotic activity of mistletoe lectin-1 by high content imaging
Source: Sci Rep. 2018 Feb 9;8:2768. doi: 10.1038/s41598-018-20915-y (PMC5807326; doi:10.1038/s41598-018-20915-y)
Supplement: Supplementary file 1 — Supplementary information [file 41598_2018_20915_MOESM1_ESM.pdf]

Supplementary information

Quantitative analysis of receptor-mediated uptake and pro-apoptotic activity of mistletoe lectin-1 by high content imaging

Beztsinna N.[1], de Matos M.B.C.[1,2], Walther J.[1], Heyder C.[2], Hildebrandt, E.[2,3], Leneweit G.[2], Mastrobattista E.[1], Kok R.J.[1*]

[1]*Department of Pharmaceutics, Utrecht Institute for Pharmaceutical Sciences, Utrecht University, The Netherlands*

[2]*ABNOBA GmbH, Pforzheim, Germany*

[3]*Institute for Mechanical Engineering and Mechanics, Karlsruhe Institute of Technology, Karlsruhe, Germany*

**corresponding author,* r.j.kok@uu.nl

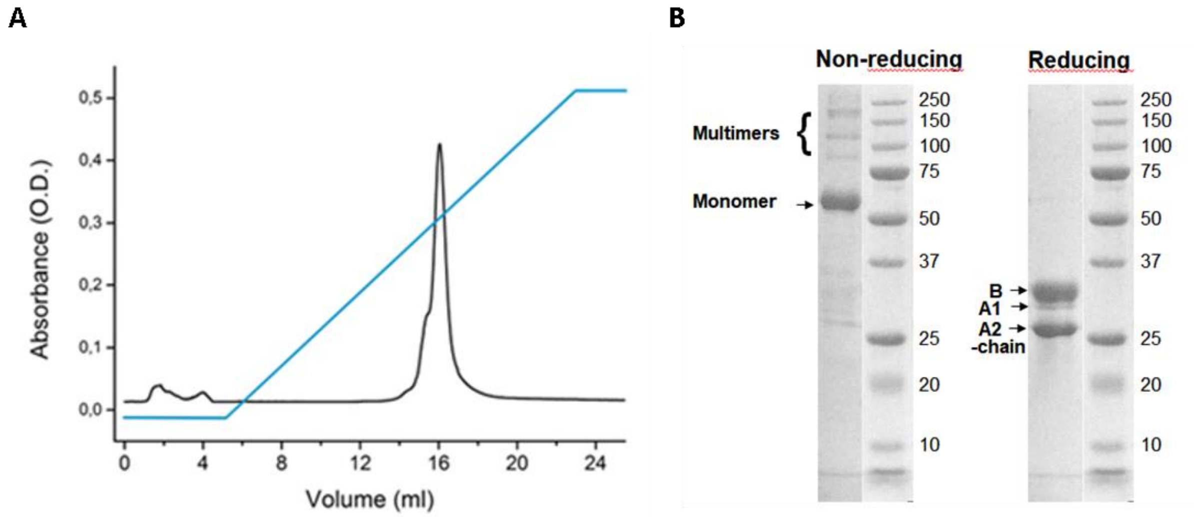

Figure S1 A. FPLC elution profile for ML1 (0.6M NaCl gradient). The main peak represents purified ML1 cytotoxin, with a small left shoulder comprising the variant (A1) of the apoptotic A chain typical for winter mistletoe harvest. B. SDS-PAGE analysis of ML1 under non-reducing and reducing conditions. Monomer ML-1 and multimers of ML-1 are observed under non-reducing conditions. Disruption of disulfide bonds under reducing SDS-PAGE conditions separates the covalently linked A and B chain of ML-1 into 26 kDa and 30 kDa fragments, respectively. A third band was detected at 29 kDa, which is a variant (A1) of the A chain found in winter harvests of the mistletoe plant.

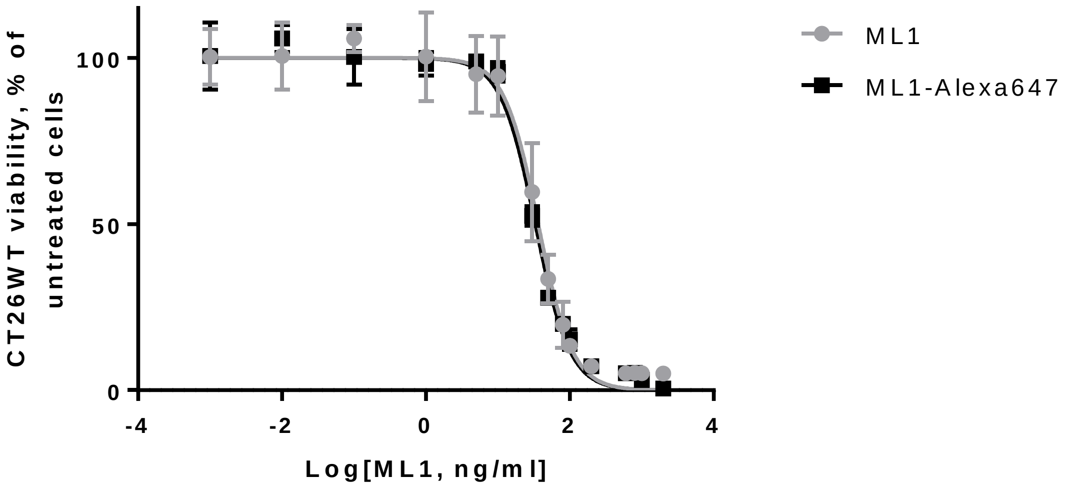

Figure S2. Comparative cytotoxicity of native and Alexa Fluor 647 labeled ML1. Respective IC50 values are 36,6 and 33,2 ng/mL.

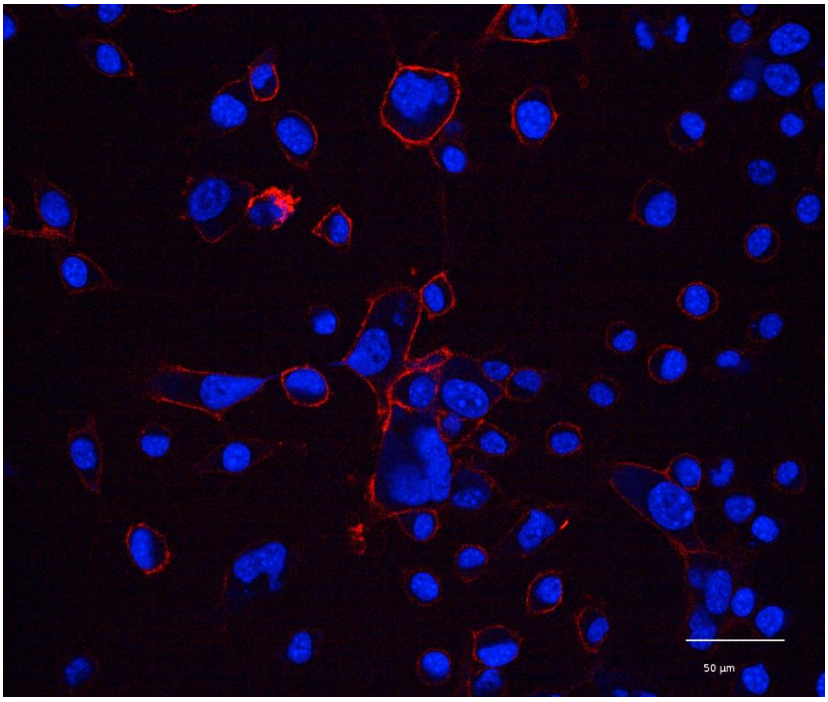

Figure S3. Confocal image of CT26 cells incubated with ML1 at 4^0C for 1h (10 µg/mL). Nuclei are stained in blue; size bar – 50 µm.

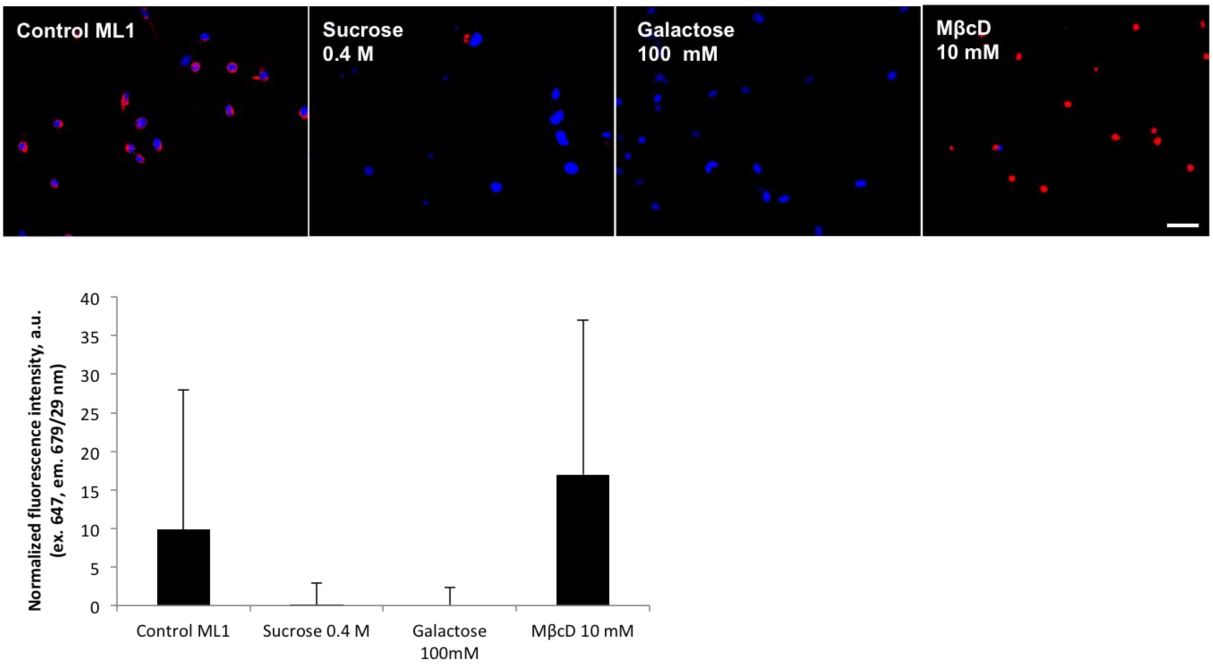

Figure S4. Confocal images of CT26 cells pre-incubated with uptake inhibitors and then incubated with ML1 for 2h (10 μg/mL) (up); Average ML1 fluorescence (n=4) in the cells quantified with Columbus suite software and normalized by subtraction of baseline cell fluorescence; nuclei are stained in blue, ML1 – red; size bar 50 μm.

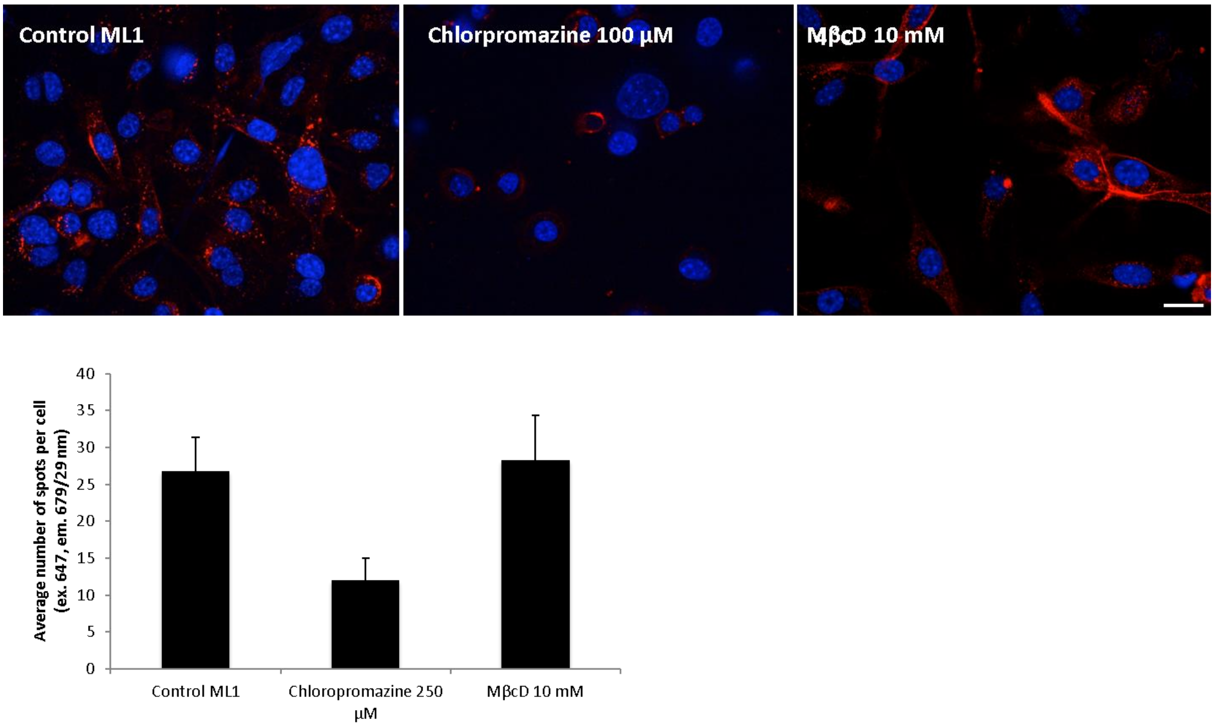

Figure S5. Confocal images of CT26 cells pre-incubated with uptake inhibitors and then incubated with ML1 for 1h (10 μg/mL) (up); Average number of spots per cell (n=3) quantified with Columbus suite software; nuclei are stained in blue, ML1 – red; size bar 20 μm.

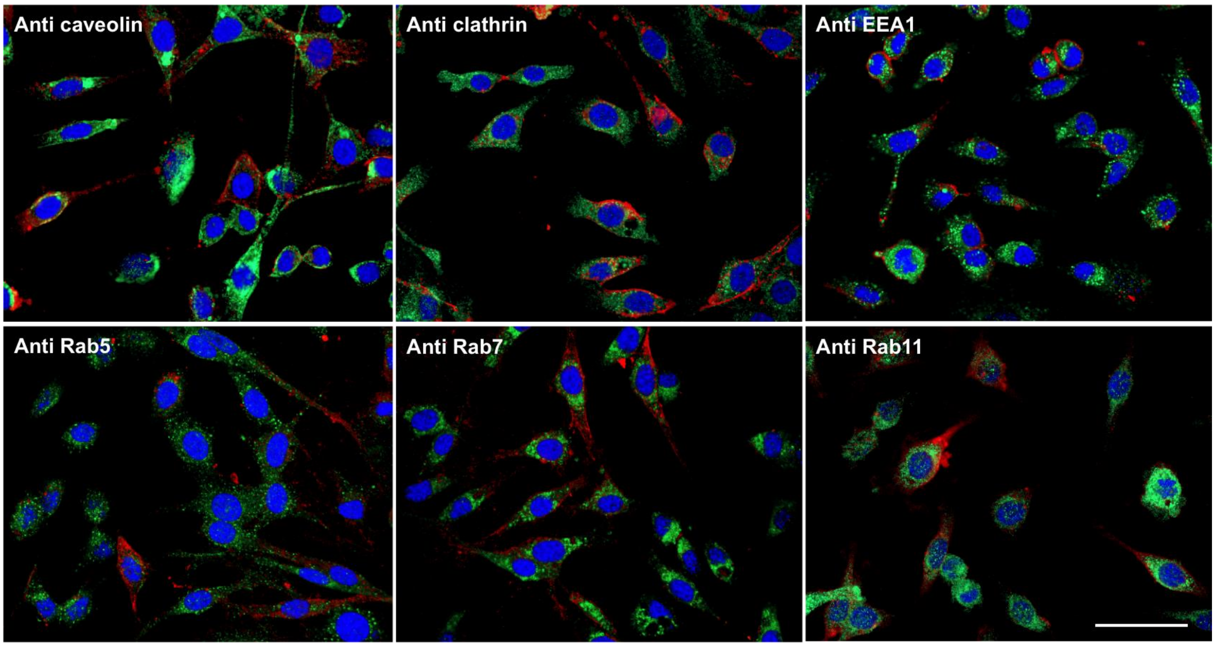

Figure S6. Confocal images of ML1 uptake (10 µg/mL, 2h) and co-staining with endo-lysosomal pathway specific antibodies; nuclei are stained in blue, ML1 – red, secondary antibody (Anti-Goat conjugated to Alexa Fluor 488) - green; size bar 50 µm.

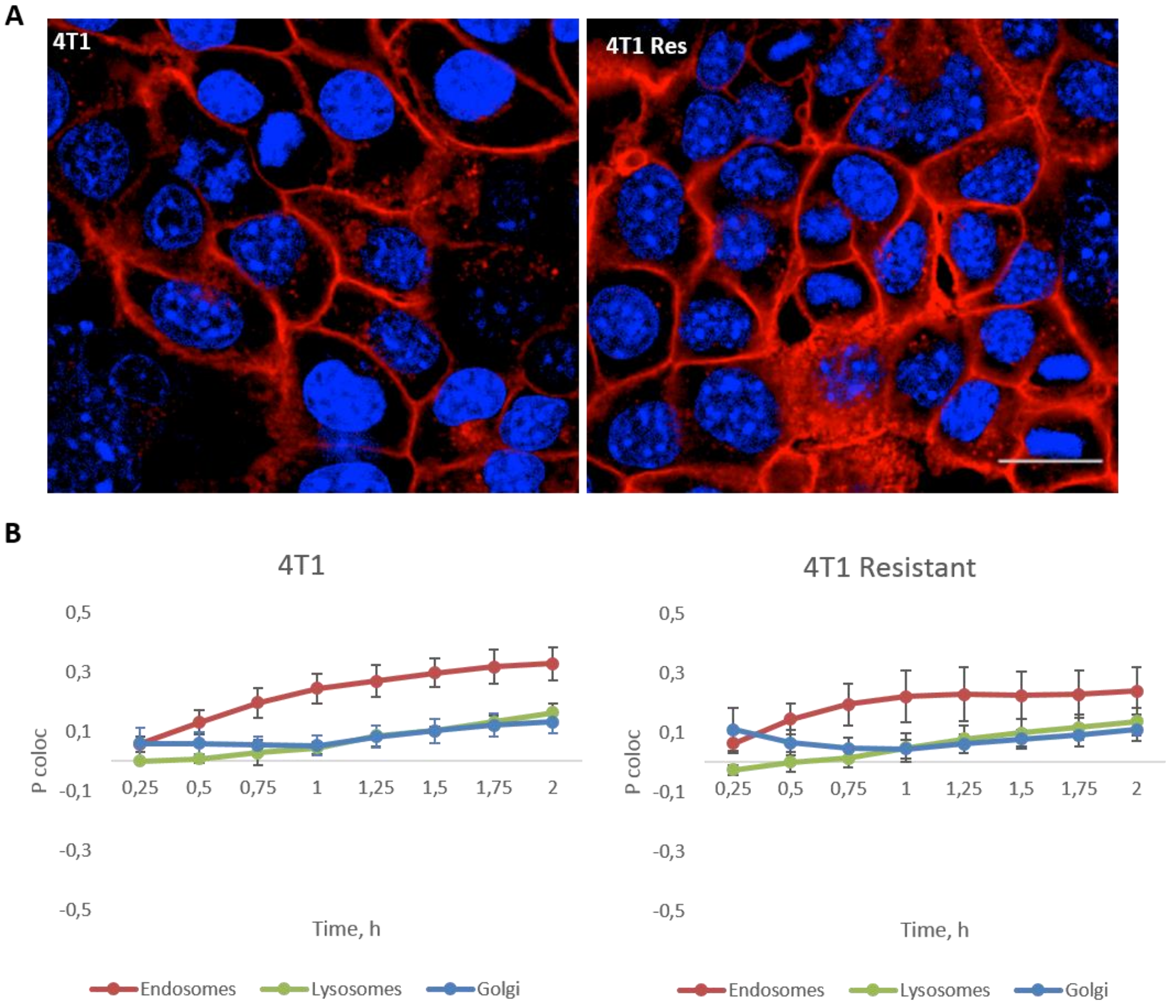

Figure S7. A. Confocal images of ML1 uptake in 4T1 control and doxorubicin resistant cells at 30 min incubation with 10 µg/mL ML1; nuclei are stained in blue, ML1 – red; size bar 20 µm.; B. Pearson correlation coefficient dynamics of co-localization ML1 with endosomes, lysosomes and Golgi in 4T1 mother and resistant cells.

Supplementary movies:

1 and 2: Time-lapse movies of ML1 induced apoptosis in CT26 cells (1) or untreated CT26 cells in the same experiment (2); nuclei are stained in blue, apoptotic cells in green (CellEvent® live staining), ML1 in red, magnification 60x. The first 8 frames of each movie are taken every 15 min for 2h and the rest 24 frames every 3 hours up to 72h.

3 and 4: Time-lapse movies of ML1 induced apoptosis in 4T1 Res cells (3) or untreated 4T1 Res cells in the same experiment (2); nuclei are stained in blue, apoptotic cells in green (CellEvent® live staining), ML1 in red, magnification 40x. In total 18 frames are taken with imaging every 4 hours up to 72h.

Table S1. Summary of fluorescent labels used for various experiments with living or fixed cells.

Target	Live-cell experiments*	Fixed cells experiments
Nuclei	Hoechst 33342	Hoechst 33342
Lysosomes	LysoTracker Green	Anti-Rab11†
Golgi	BODIPY TR C5 -ceramide complexed to BSA	-
Endosomes early	pHrodo Green Dextran conjugate	Anti-EEA1, Anti-Rab5
Endosomes late	-	Anti-Rab7
Cell membrane/ protein transport	Wheat Germ Agglutinin Oregon Green conjugate	-
Caveolin-mediated uptake	Cholera toxin subunit b Alexa Fluor 488 conjugate	Anti-caveolin
Clathrin-mediated uptake	-	Anti-clathrin
Apoptotic cells	CellEvent caspase-3/7 Green Detection Reagent	CellEvent caspase-3/7 Green Detection Reagent

* All live cell labeling reagents and fluorescent dyes were purchased from Thermo Fisher Scientific, Naarden, The Netherlands.

† Endosomal Marker Antibody Sampler Kit #12666 was purchased from Cell Signaling Technology Europe, B.V., Leiden, The Netherlands.
